# Changes in relationships, HIV risk, and feelings towards PrEP: findings from a qualitative explanatory study among participants in the CHARISMA intervention trial

**DOI:** 10.1186/s12905-023-02603-w

**Published:** 2023-08-22

**Authors:** Miriam Hartmann, Noah Triplett, Sarah T. Roberts, Michele Lanham, Krishnaveni Reddy, Siyanda Tenza, Nonkululeko Mayisela, Dorica Mbewe, Ontathile Maboa, Lydia Mampuru, Elizabeth E. Tolley, Thesla Palanee-Phillips, Elizabeth T. Montgomery

**Affiliations:** 1 Women’s Global Health Imperative, RTI International, Berkeley, California USA; 2 Department of Global Public Health, Karolinska Institutet, Stockholm, Sweden; 3 FHI360, Durham, USA; 4 Wits RHI, Faculty of Health Sciences, University of the Witwatersrand, Johannesburg, South Africa; 5 Department of Epidemiology, School of Public Health, University of Washington, Seattle, USA

**Keywords:** Intimate-partner violence, Pre-exposure prophylaxis, HIV prevention, South Africa, Adult women, Empowerment-based counselling intervention

## Abstract

**Background:**

Intimate partner violence (IPV) and other relationship-based challenges have been demonstrated to reduce women’s ability to use pre-exposure prophylaxis (PrEP) effectively for HIV prevention. The Community Health Clinical Model for Agency in Relationships and Safer Microbicide Adherence (CHARISMA) intervention was designed to mitigate these challenges and increase South African women’s agency to use PrEP. The CHARISMA randomized controlled trial did not identify statistically significant differences in PrEP adherence or relationship dynamics between the intervention and control arms. As such, the aim of this explanatory qualitative sub-study was to understand women’s experiences with the CHARISMA trial and explore reasons for the null results.

**Methods:**

Twelve CHARISMA trial participants were purposively selected to participate in serial in-depth interviews, which took place at the trial end and 3 months later. Participants represented individuals who had received each of the three counselling modules, 1) healthy communication counselling, 2) PrEP disclosure counselling, or 3) IPV counselling, as well as those in the control arm who received IPV standard-of-care counselling.

**Results:**

A thematic case analysis revealed numerous positive relationship outcomes among intervention participants, including identifying and ending unhealthy relationships, gaining a sense of personal empowerment, and enacting more positive behaviors and HIV risk reduction strategies in subsequent relationships. These positive shifts were occasionally described as contributing to decisions to discontinue PrEP use, which may partly explain the limited impact of the intervention on PrEP adherence.

**Conclusions:**

Future investigations of counselling interventions addressing relationship-based barriers to PrEP use should account for changing risk dynamics and need for PrEP.

## Introduction

An estimated 26% of South African women are living with human immunodeficiency virus (HIV) [1]. Though significant efforts have been undertaken to reduce the transmission of HIV in South Africa, including efforts to increase access to pre-exposure prophylaxis (PrEP) [2], HIV remains prevalent and disproportionately impacts women [1]. The disproportionate burden of HIV among women in South Africa has been attributed in part to gender-based and intimate partner violence (IPV) [3]. IPV is the most common type of violence targeting women [4], and it has been linked to HIV transmission through both forced sex with a partner [5] and limited ability to enact behaviors to reduce HIV, such as taking PrEP [6]. Given these dynamics, research has increasingly focused on understanding how to best support women to safely and consistently use PrEP and reduce incidence of IPV [7–9].

The Community Health Clinical Model for Agency in Relationships and Safer Microbicide Adherence (CHARISMA) intervention was designed specifically to mitigate relationship challenges and increase agency to use PrEP in South African women, thereby addressing multiple factors that influence their risk of HIV acquisition [8]. The CHARISMA intervention was designed in collaboration with partners in South Africa and guided by a theory of change that linked counseling modules to specific mechanisms of change (see [8] for more information on intervention development and methods for the theory of change). The CHARISMA intervention was evaluated in a randomized controlled trial (RCT) that compared the CHARISMA intervention against a robust standard of care that included PrEP provision and basic empathetic counseling using the WHO LIVES approach [10]. There were no clear main effects of the CHARISMA intervention on PrEP adherence or experiences of IPV [10]; however, there was evidence that women’s mental health improved across trial conditions [11]. These results have prompted further analysis of the CHARISMA trial to better understand findings and optimize future efforts to intervene upon IPV and improve PrEP use.

Qualitative research complements quantitative data collection in RCTs, offering the potential to explore more complex aspects of the intervention and its implementation with greater depth [12]. Qualitative data may be particularly useful for trials of behavioral interventions, as it can provide information about the “active ingredients” of interventions and offer opportunities to explore intervention effects among subgroups of participants [13]. Further, for studies without clear main effects, qualitative research may offer opportunities to explore any potential reasons for the lack of effects [14].

**Fig. 1:**
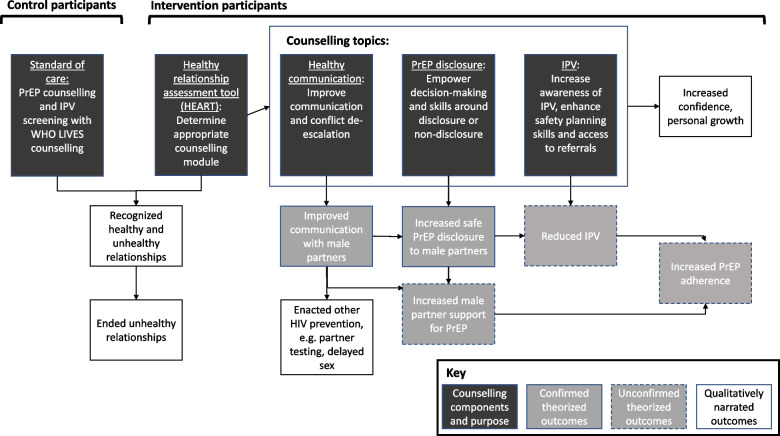
CHARISMA intervention theory of change with additional qualitatively narrated outcomes

The aim of this qualitative sub-study was to understand women’s experiences with the CHARISMA trial and explore potential mechanisms of change with the CHARISMA intervention. We focused specifically on exploring these experiences and mechanisms according to the CHARISMA intervention theory of change (presented in Fig. 1 and described below). Given the main trial’s null findings in relation to women’s PrEP adherence and reduction in intimate partner violence, the analysis also focused on explaining what may have contributed to these findings for women over time. We aimed to refine our understanding of what works and for whom in intervening upon IPV and improving PrEP adherence.

## Methods

### Main CHARISMA trial design and intervention

#### Design

The CHARISMA trial was a two-arm, randomized (1:1), controlled study of a behavioral intervention—CHARISMA—to reduce social harms (SHs) and intimate partner violence (IPV), increase healthy relationship dynamics, and improve oral PrEP adherence (ClinicalTrials.gov registration id: NCT04092114). The trial enrolled 407 women from a densely populated urban neighborhood in Johannesburg, South Africa who had to be ages 18–45, HIV-negative and interested in using PrEP for HIV prevention, have a current sexual partner, be sexually active, and have no previous history of participating in clinical trials or longitudinal studies on HIV prevention to be eligible for trial enrollment. Enrollment occurred from October 2018- October 2019. Participants were dispensed with oral PrEP and followed for a period of six-months. Study visits included screening, enrollment, and months 1, 3, and 6 of follow-up. At months 3 and 6, behavioral questionnaires were administered where participants were asked about sexual relationships and behavior, and experience of violence. Dried blood spots (DBS) were collected at each timepoint to assess PrEP adherence and analyzed following the study as part of the primary study outcomes [15]. At month 6/study exit participants were offered a 3-month supply of PrEP and linked with available PrEP providers for continued use.

#### Intervention

At enrollment, participants were randomized in a 1:1 ratio to receive either the CHARISMA intervention (intervention arm) or standard of care (control arm). The CHARISMA intervention included a lay counsellor-administered relationship assessment tool called the HEAlthy Relationship Assessment Tool (HEART), followed by targeted empowerment-based counselling delivered upon enrollment with a follow-up session at month 1. All intervention participants received counselling in healthy relationships, as well as counselling on either 1) general partner communication and relationship skills, 2) partner disclosure and communication around PrEP, or 3) responding to IPV and safety planning. The choice for the follow-on topic was guided by an algorithm embedded within the HEART delivery, which assessed factors such as risk of IPV and whether a woman had disclosed PrEP use to her partner. Figure 1 displays the intervention theory of change in terms of how the counselling topics were theorized to increase PrEP support and reduce relationship-based barriers to PrEP use, such as experience of IPV.

Control arm participants received standard of care PrEP counselling, as well as basic IPV screening followed by first-line support using the WHO LIVES approach to counselling and referrals to external support. In brief, the LIVES approach includes: Listening to women, Inquiring about their experiences, Validating their emotions, Ensuring safety, and Supporting through referrals [16]. All participants also received access to ‘male partner packets’, which offered materials on PrEP to be shared with male partners with ‘opt-out’ delivery for intervention arm participants and ‘opt-in’ delivery for control-arm participants. Additional details on the development and content of the HEART and counselling intervention, as well as trial procedures and results are described elsewhere [8, 10, 17, 18].

#### Qualitative component

A subset of 12 participants were purposively selected for serial in depth interviews (IDIs) conducted after exiting the trial (at months 6 and 9). Selection was based on study arm assignment and counseling exposure. We included equal numbers of intervention participants who had received each of the three counselling modules, i.e. 1) healthy communication counselling, 2) PrEP disclosure counselling, or 3) IPV counselling (*n* = 3 per counselling topic). Three additional participants in the control arm who received either IPV standard-of-care counselling, or other counselling for potential social harms, were purposively selected to explore differences in counselling topics and approach with those from intervention arm participants and to assess any potential “intervention contamination” or exposure among control arm participants. A sample size of 3 per subgroup was chosen because of recommendations that 3 participants per subgroup within a nested sample allows for adequate information power [19]. Participants were recruited to take part in IDIs at their month 6/study exit visit and underwent a separate informed consent process to participate. IDIs were conducted in the language of the participant’s choosing—either English or isiZulu—by an experienced qualitative researcher of the same sex. They were conducted in a private space to ensure confidentiality and were audio recorded for later transcription directly into English (when appropriate). Month 6 interviews explored study experiences, sexual partner relationships, PrEP support and use, intervention experiences and any partner relationship, PrEP use, or other life changes related to CHARISMA (intervention participants only), and/or experiences with violence counselling (among control and intervention participants). The month 9 interviews covered similar topics, specifically exploring changes since the last interview in key domains, such as sexual partner relationships, PrEP use, and application of learnings from CHARISMA or other counselling experiences.

#### Analysis

IDI transcripts were coded and analyzed using Dedoose [20] by a team of four social science analysts from the US and South Africa. Intercoder reliability for Dedoose coding was established using coding tests in Dedoose. Coders had an average kappa score of 88%, indicating high reliability. Analysis of data employed an approach whereby coded qualitative content was exported from Dedoose and along with some quantitative data points were summarized for each participant, inclusive of each timepoint for which they were interviewed, as a case memo using a standard template. The case memos included the following: arm assignment, PrEP adherence data from dried blood spot specimens, self-report of violence during the study, visit timepoint (month 6 or 9), and themes related to the intervention content (see Fig. 1) and the parent study outcomes (adherence, violence, relationship well-being). More specifically, themes were generated from coded data and illustrative quotes at each IDI time point related to sexual relationships, motivation to join or stay in CHARISMA, PrEP use, experience with counselling, and outcomes related to relationships and PrEP use, and any other emergent topics or behavioral patterns. Finally, case memos were further reviewed as sets according to their specified subgroup and a final analysis memo documented patterns across subgroups. All case memos and summary memos were reviewed and discussed during team meetings to ensure the reliability of the reported themes. Any disagreements were resolved through consensus.

The CHARISMA study, including the qualitative component, was reviewed and approved by the Human Research Ethics Committee at the University of Witwatersrand and overseen by the Institutional Review Board at Research Triangle Institute (RTI) International.

## Results

### Participant characteristics

A total of 12 participants were involved in the qualitative study, 9 of whom completed both interviews. Four did not complete their month 9 interviews due to unavailability (2 from control arm, 1 PrEP disclosure, and 1 IPV counselling participant). Three participants were from the control arm, 2 of whom received IPV-related SOC counselling, and the remaining 9 were intervention participants during the CHARISMA trial. Intervention participants were evenly spread across those who received healthy communication counselling (*n* = 3), PrEP disclosure counselling (*n* = 3), or IPV counselling (*n* = 3).

At the time of baseline enrollment into CHARISMA, participants in the qualitative sub-study were on average 27 years old, all had completed some secondary school, close to half (42%) were food insecure, and only one lived with her partner. Approximately one-third reported experiencing IPV from their current partner at baseline, and two-thirds reported partner awareness of their PrEP use or interest. Only one third reported feeling at risk for HIV, although all reported an HIV risk factor or circumstance, which was defined as lab-diagnosed STI or report of inconsistent condom use, belief or knowledge that primary sex partner has other partners, transactional sex in past 30 days, > 1 sex partner in last 30 days, or unknown partner HIV status. Only one participant reported IPV at any of the follow-up visits. This was a control arm participant who received IPV SOC counselling. In terms of adherence, DBS results were categorized into three groups: 1) persisted, which was defined as having medium or high adherence at their month 6 drug result (i.e. drug levels corresponding to ≥ 4 oral doses/week); 2) minimal/non-use, which was defined as having low (i.e. drug levels corresponding to < 4 doses per week) or missing adherence results at both month 3 or 6; and 3) discontinued, which was defined as having high or medium adherence at month 3 and low adherence at month 6. Based on this stratification, 4 participants were categorized as ‘persisted’ PrEP use, 4 had ‘minimal/non-use’ of PrEP throughout the trial, and 4 ‘discontinued’ PrEP use. See Table 1.

**Table 1:** Participant characteristics at enrollment (baseline) and during follow-up

Characteristic	Total, N (%) (*N* = 12)
***Socio-demographics, IPV experiences and HIV attitudes at baseline***	
Qualitative study completion status	
Completed 1 IDI	12 (100%)
Completed 2 IDIs	8 (67%)
Age (median, range)	27.5 (21–45)
18–24 years	1 (8%)
25 + years	11 (92%)
Completed some secondary school	12 (100%)
Food insecurity sometimes or often, past month	5 (42%)
Married or cohabitating with partner	1 (8%)
Any IPV by current partner, past 3 months	4 (33%)
Partner aware of PrEP use or interest in PrEP	8 (67%)
Any HIV risk perception	4 (33%)
Any HIV risk behavior/circumstance^a^	12 (100%
***IPV experiences and adherence behavior during follow-up***	
IPV reported at follow-up surveys	1 (8%)
Adherence category (DBS)^b^	
Persisted	4 (33%)
Minimal/non-use	4 (33%)
Discontinued	4 (33%)

^a^At baseline, has lab-diagnosed STI or reports inconsistent condom use, thinks or knows partner has other partners, transactional sex in past 30 days, > 1 sex partner in last 30 days, or unknown partner HIV status

^b^Adherence categories: Persisted was defined as having medium or high adherence at their month 6 drug result; minimal/non-use was defined as having low or missing adherence results at both month 3 or 6; discontinued was defined as having high or medium adherence at month 3 and low adherence at month 6

### Qualitatively narrated mechanisms of change

As demonstrated in Fig. 1, qualitative interviews with participants revealed several areas where theorized mechanisms of change were confirmed, as well as contributed new mechanisms of change. These broadly fell into the categories of changes related to sexual relationships and HIV risk (new), choices around PrEP disclosure and ability to respond to male partner concerns (confirmed), and enhanced confidence and personal growth (new). The following sections describe these themes and sub-themes, calling attention to differences by study arm or counselling category, where noted.

### Sexual relationship changes and HIV risk

Some of the most substantial shifts narrated by participants were changes in their relationship dynamics with male partners, although with notable differences described by intervention vs. control arm women. Broadly, these changes included ending unhealthy relationships, improving communication and conflict negotiation skills, and enacting HIV risk reduction skills, and are presented by these three themes below.

#### Ending unhealthy relationships

Half of the participants interviewed, representing cases from each of the intervention counseling categories and the control arm, reported ending relationships during the study period. All of these relationships were described as unhealthy and riddled with problems such as male expression of infidelity and controlling behaviors. While all cases described learning what was healthy and unhealthy in relationships at the CHARISMA trial, cases from the different intervention counseling categories described how this awareness contributed to their decisions to end relationships.

*“I think it made me realise what's normal and what’s not normal and to be truthful about it you know, and just not to say okay I know that’s abusive and then you keep quiet about it. If something is not okay it is not okay, period*.” (Communication counselling case, minimal/non-use)

“*Yes, I ended up realizing that even when I talk to him, with the questions that they ask me here in the study that I can walk away, he is not the only person, there are other people out there who can love me.”* (IPV counselling case, minimal/non-use)

### Improving communication and conflict negotiation

Cases receiving all intervention counselling types also reported improved communication and conflict negotiation skills in their sexual relationships. These changes were described as occurring in both new and ongoing partnerships. Among the communication counselling participants, there was a greater emphasis on the value of communication in one’s relationship and a demonstrated use of skill-building activities covered in the counselling. One woman described using an exercise, that was conducted individually in counselling, subsequently with her partner to reflect on their current relationship and where they want to be in the future.

“*So, in the last video that I was watching for you to build the trust and communication you have to sit down and face your partner…write what you like and what you dislike about the relationship, and then work on them. So, after joining the study we did that. Then we discovered that most of the things we like are common. Then what didn't happen at first, it's that we had (too much) pride to talk, to challenge each other. Because in a relationship, you have to challenge each other and see the way forward, because it's not about you having sex all the time, but some of the personal things like where do you see yourself in 5 years’ time, the goals; you have to have goals. So now I know what he wants, and he knows what I want.”* (Communication counselling case, persistent use)

Conflict de-escalation was also a skill that both communication and IPV counselling participants described utilizing in their relationships, in particular, pausing a discussion when tension felt high. At least one participant, however, described how this was hard to enact, both when one has engrained communication patterns with a partner and also during times of high stress such as the COVID lockdowns and economic pressures she faced.

*“It was hard at first because, remember, if you are in a relationship for a very long time and you are used to the way you are arguing, and your habits. Now to leave them and starting to use another route at first it was very hard especially when I had to control my anger. Or when the argument starts, I must tell myself okay do not say this, try to say this. Sometimes I would want to use the old way to finish the fight fast, yeah but now it has helped me, because I have gotten used to it.”* (Communication counselling case, minimal/non-use)

Contrary to intervention participants, control arm participants more often described ongoing mistrust and unhealthy relationship patterns with their partners. This was even in cases where participants described trying to enact conflict de-escalation skills, such as the case of one woman who described a change in her willingness to apologize to de-escalate conflict, which she says she learned from the study.

*“A research assistant …She explained that I was not [in] a healthy relationship, [my partner and I] must talk, and [I should] ask for forgiveness...For me it was difficult to do that, but now I can talk to him. I was apologizing over the phone, but he would not accept my apology and [said] my apology means nothing.”* (Control, IPV SOC case, discontinued use)

### Enacting HIV risk reduction in new relationships

Upon starting new relationships, a number of intervention-arm women described having open conversations about both their emotions and what they wanted in terms of HIV prevention. Several described testing for HIV with their new partners. This theme was not reported by the qualitative sample of control-arm participants.

*“We've tested for HIV and AIDS and then we had negative results. And then I told him about PrEP…So that was the skill that I used in this relationship that I've been into with my new partner.”* (PrEP disclosure, discontinued use)

And a couple of women described being empowered to negotiate delaying sex with new partners until more trust was built. One participant explained how she felt comfortable and confident refusing sex from a new partner who did not want to use condoms when she suggested their use on the phone with him. She added,

“*I would say I have been wise because this man is always talking about sex which means he is only interested in having sex with me, so such a person might be a user. You can see that he just wants to use women. That’s what he wants from them.”* (PrEP disclosure, persistent use)

### PrEP disclosures, responses, and discontinuation

#### PrEP disclosure

Almost all women reported disclosing PrEP use to at least one partner in their life and many also disclosed to other family or community members. The exception to this is that women who received IPV counselling, often reported fearing their partners’ reactions to PrEP and thus did not disclose. Among those who did not disclose PrEP use to their partners, one woman described how this contributed to her stopping PrEP use as she faced challenges with hiding it. However, similar to what was described above among other participants, she also suggested that she was no longer at risk of HIV after testing with her partner.

“*Maybe the day would come when I tell myself that I would use PrEP again because it is not safe out there. Maybe one day I would need it, one day when I am at risk …The reason is that I have noticed changes in my partner [is]…we sat down and talked about the importance of HIV/AIDS, and that he should go and do the tests, so he went and found out that he is negative. That is the reason I stopped continuing with it (PrEP).”* (IPV counselling case, discontinued use)

#### Response to PrEP disclosure

Participants who disclosed PrEP use described mixed reactions from male partners and family members, with a number of male partners raising questions of trust, unfaithfulness, or HIV status as potential reasons for PrEP use, but none using physical violence. Women who had received PrEP disclosure counselling more often described successfully responding to these concerns and gaining support from their partners. As one participant explained in this conversation with her partner, she rationalized her use due to external sources of risk, which helped earn his understanding:

*“At first, he was reluctant because he was telling himself that I want to take PrEP for cheating on him but after I explained to him it’s not so, it’s not based on me wanting to cheat on him. Anything can happen. I could get raped or anything, so at least you would know that even if anything bad happens, you will know that you are safe.” (PrEP disclosure case, persistent use)*

Several women also described how telling partners/family about side effects led to less support upon disclosure. For at least two women (from the control and communication counselling arms, respectively), external concerns subsequently induced their own internal debate about whether to persist with PrEP use. For example, one control arm participant described how when she disclosed use to her family and shared about the rash she was experiencing, her mother and siblings expressed a lack of understanding of why she continued to take PrEP. After their reaction, the participant said she “*felt like maybe they are right, PrEP wasn’t good for me. It didn’t feel good in the beginning*.” (Control arm case, persistent use).

#### Reasons for PrEP discontinuation

Those who discussed their discontinuation from taking PrEP during the 6-month CHARISMA study described doing so for a variety of reasons, which included dynamics with partners and life circumstances. These included no longer feeling at risk after HIV testing or relationship changes, being pregnant or having a desire to get pregnant and associated fears that PrEP would be dangerous, as well as experiencing other life stress that created pressure in one’s life and relationships. For the latter, it’s relevant to note that many of these interviews were conducted during the early months of COVID-19 restrictions, and as one woman put it, life was generally stressful and accessing PrEP viewed as another stressor.

*“The most challenging part for me to use PrEP is the stress that I am experiencing now … PrEP feels like another burden now. Its feels like another responsibility.”* (Communication counselling case, minimal/non-use)

#### Other challenges with PrEP adherence

As indicated by the DBS results, PrEP adherence for 6 months was challenging for many, and in addition to the partner-related challenges noted in the previously described results, non-partner related challenges were faced. Difficulties reported were largely similar across participant counseling categories, the majority described side effects, as well as occasional logistic challenges with taking PrEP, such as when they had functions to attend or when drinking on the weekends. And finally, in terms of continued PrEP use after the trial through the South African public health clinic settings, almost all the women, except the IPV counselling cases, expressed interest in continuing PrEP. However, stigma in these public clinic settings was seen as a barrier. One woman described facing ill treatment from providers who ‘questioned’ her PrEP use when she sought PrEP in a public clinic after the trial.

### Personal growth

The final notable theme present in these data, which was evident only within the narratives of intervention arm participants, was that of personal growth and empowerment. This theme was described by almost every intervention participant and explained as improved self-esteem, confidence, and general empowerment to “speak up” in various aspects of their lives. Participants felt enhanced freedom to communicate to partners based on skills learned, and described the downstream secondary intervention effects of resulting in improved relationship dynamics. As one woman explained,

“*I think the support I get from my partner is enabling me to have self-confidence and be able to talk, like to be free.”* (IPV counselling case, discontinued use)

Personal growth was also displayed in terms of confidence to talk specifically about HIV prevention with partners:

*“Because I was able to talk about things I never spoke about, like I never spoke to my partner, even the father of my first child, I never spoke to him about things like HIV testing, even that I don’t want to have sex or use a condom…I have never. Some of the things I wouldn’t have said them because I felt like…I didn’t know how to start talking about them.”* (IPV counselling case, minimal/non-use)

## Discussion

Findings from this nested qualitative study among CHARISMA trial participants revealed numerous positive relationship outcomes among intervention participants as compared to controls, including identifying and ending unhealthy relationships, gaining a greater sense of personal empowerment, and enacting more positive behaviors and HIV risk reduction strategies subsequent relationships. These shifts were occasionally described as contributing to decisions to discontinue PrEP use, which may partly explain the limited impact of the intervention on PrEP adherence [10]. While described relationship changes didn’t correspond to measurable reductions in IPV in the trial, these results are encouraging, as they suggest that the empowerment-based counseling addressed topics that were important to the women and helped them build skills that promoted healthier relationships, lower HIV risk, and enabled them to make their own decisions about PrEP use continuation.

In addition to their explanatory role, these findings are important for their role in contributing to refining an intervention theory of change, counselling practices, and outcome measurement in future trials of relationship-based counselling for PrEP users. The shifting motivation and need for PrEP use among women enacting positive relationship changes should be accounted for in an intervention theory of change and in PrEP use provision, more broadly. The concept of ‘seasons of risk’ is one that has been in the PrEP world for a number of years [21, 22], however accurate measurement of risk or even assessment of risk among women themselves is still challenged and may not always align with epidemiological risk [23–27]. This misalignment consequently challenges tailoring PrEP recommendations and counselling based on risk. While our counselling focused on empowering women to make their own decisions about, for example, disclosing PrEP use or improving their relationship communication, trust, and general health, our theory of change did not account for the possibility that women’s decisions may reduce, rather than increase, PrEP use. Other quantitative trial measures around relationship changes, and endings, may also have been inadequate in capturing critical shifts in trust, or other qualities of communication changes that were described by trial participants and may factor in their risk assessment. Finally, the high levels of reported relationship dissolution was surprising for a population of adult women who were expected to be in more stable relationships and may also be important for future counselling programs to address. CHARISMA counselling was designed to be administered whenever a woman indicated she had a new partner, regardless of when it was during the trial. This may have supported intervention arm women to enact learned ideas about healthy relationship qualities as described by these participants.

While there seemed to be clear differences in relationship health and HIV risk reduction behaviors between intervention and control arm participants, we did see some indications of intervention contamination, as control participants reported receiving counselling on healthy relationships, communication, and conflict negation even if not successfully implemented. While the trial had numerous measures in place to minimize and measure contamination, including using different counsellors for intervention vs. control counselling and periodic structured observations of counselling sessions, all trial staff were highly trained in gender equitable care and gender-based violence, which may have contributed to contamination. Control arm participants in the trial received LIVES counselling, which focuses on inquiring, validating, and supporting someone, and it may have felt natural for counsellors to offer advice or comment on healthy relationships in this context. That said, as qualitatively described by this sample of women, however, it seems that CHARISMA counselling provided additional “actionable” skills to more directly make changes in their relationship dynamics therefore supporting the added value of CHARISMA to an approach like LIVES.

Finally, women did note other challenges to PrEP use and access that were not driven by relationships. These barriers, including side effects, stigma associated with PrEP use, and logistical challenges with taking PrEP, have been demonstrated in numerous other studies [28–30] and point toward an ongoing need for multilevel interventions to address family related barriers, perceived or experienced clinic stigma, and better support for side effects.

No study is without its limitations. For one, our study sample size was small and not generalizable to a broader population or even to the trial population. However, we did take care to ensure the sample was purposive in a way that we felt would capture a variety of experiences with the trial and we were able to see common themes indicating some level of saturation. Additionally, the longitudinal design of the study and our case analysis approach strengthened our ability to dive deeply into each individual’s circumstances, and to evaluate their individual stories in relation to the key variables of exposure (counseling receipt, relationship circumstances) and outcomes of PrEP use and IPV and relationship consequences over time. Another limitation to this study, however, was the uneven completion of both interviews by participants. Of the four participants who did not complete a second interview, two were from the control arm, which may indicate an important distinction in study experience. Finally, while we chose not to interview male partners given the intervention’s focus on empowering women to make their own choices about their relationships and PrEP use, we are missing information from male partners to understand their experiences of relationship change and HIV risk reduction, or lack thereof, from their female partners.

## Conclusion

In the context of rapidly expanding PrEP implementation worldwide and PEPFAR requirements to screen for and address IPV within PrEP delivery, interventions such as CHARISMA offer a structured approach for providers to address not only IPV, but also other relationship dynamics that may influence PrEP use. Qualitative stories of change from CHARISMA participants support the value of empowerment-based counselling on women’s ability to identify and enact healthy relationship behaviors and HIV risk reduction strategies. When used in combination with other strategies addressing additional barriers to PrEP use, an approach that centers women’s wellbeing around their empowerment and autonomy to choose what works best for them, we believe women can be better supported in their HIV prevention efforts.

## Data Availability

The datasets used and/or analysed during the current study are available from the corresponding author on reasonable request.

## Abbreviations

CHARISMA: Community Health Clinical Model for Agency in Relationships and Safer Microbicide Adherence
DBS: Dried blood spots
HEART: HEAlthy Relationship Assessment Tool
IDI: In-depth interview
IPV: Intimate partner violence
LIVES: Listening to women, Inquiring about their experiences, Validating their emotions, Ensuring safety, and Supporting through referrals
GBV: Gender-based violence
HIV: Human immunodeficiency virus
PrEP: Pre-exposure prophylaxis
RTI: Research Triangle Institute
SH: Social harms
SOC: Standard of care
WHO: World Health Organization
